# Regional differences in trait-like characteristics of the waking EEG in early adolescence

**DOI:** 10.1186/1471-2202-14-117

**Published:** 2013-10-09

**Authors:** Dominik C Benz, Leila Tarokh, Peter Achermann, Sarah P Loughran

**Affiliations:** 1 Institute of Pharmacology and Toxicology, University of Zurich, Zurich, Switzerland; 2 Department of Psychiatry and Human Behavior, Alpert Medical School of Brown University, Providence, USA; 3 Zurich Center for Integrative Human Physiology, University of Zurich, Zurich, Switzerland; 4 Neuroscience Center, University and ETH Zurich, Zurich, Switzerland; 5 School of Psychology, Illawarra Health & Medical Research Institute, University of Wollongong, Wollongong, NSW, Australia

**Keywords:** Spectral analysis, Development, Endophentoype, Clustering, Alpha activity

## Abstract

**Background:**

The human waking EEG spectrum shows high heritability and stability and, despite maturational cortical changes, high test-retest reliability in children and teens. These phenomena have also been shown to be region specific. We examined the stability of the morphology of the wake EEG spectrum in children aged 11 to 13 years recorded over weekly intervals and assessed whether the waking EEG spectrum in children may also be trait-like. Three minutes of eyes open and three minutes of eyes closed waking EEG was recorded in 22 healthy children once a week for three consecutive weeks. Eyes open and closed EEG power density spectra were calculated for two central (C3LM and C4LM) and two occipital (O1LM and O2LM) derivations. A hierarchical cluster analysis was performed to determine whether the morphology of the waking EEG spectrum between 1 and 20 Hz is trait-like. We also examined the stability of the alpha peak using an ANOVA.

**Results:**

The morphology of the EEG spectrum recorded from central derivations was highly stable and unique to an individual (correctly classified in 85% of participants), while the EEG recorded from occipital derivations, while stable, was much less unique across individuals (correctly classified in 42% of participants). Furthermore, our analysis revealed an increase in alpha peak height concurrent with a decline in the frequency of the alpha peak across weeks for occipital derivations. No changes in either measure were observed in the central derivations.

**Conclusions:**

Our results indicate that across weekly recordings, power spectra at central derivations exhibit more “trait-like” characteristics than occipital derivations. These results may be relevant for future studies searching for links between phenotypes, such as psychiatric diagnoses, and the underlying genes (i.e., endophenotypes) by suggesting that such studies should make use of more anterior rather than posterior EEG derivations.

## Background

The EEG can be recorded non-invasively and inexpensively, with accurate estimates of cortical oscillations made with just a few minutes of recording. EEG recordings are of great utility not only for learning about the healthy brain, but also reflect changes in brain structure/function associated with psychiatric disorder. The heritability and stability of the EEG add to its utility. For example, studies of twins have found that the human waking EEG is one of the most heritable traits with heritability estimates ranging between 0.55 and 0.9 [1,2], dependent mainly on frequency band [3-5] and age [6,7]. Heritability estimates are also region specific, with studies generally finding higher heritability over posterior as compared to anterior regions [4,5,8].

In addition to being highly heritable, the waking EEG spectrum is stable across time. A number of studies in adults, adolescents, and children have shown high test-retest reliability between sessions (separated in time by several months and even up to several years) for both absolute and relative spectra [3,9-11]. Mirroring the heritability studies, reports on the stability of the EEG spectrum have also shown greater stability in posterior compared to anterior regions [12]. These studies have made claims of stability primarily by computing correlation coefficients between recordings [9-11,13,14] or by ANOVA analysis [15] within a given frequency band. One limitation of correlation measures is that although they are an indication of the stability of band power within an individual, they do not address how unique power in a band is compared to that of other individuals. In contrast, intraclass correlation coefficients (ICCs) take into account both the within and between subject variability, with high ICC values indicating that a feature is stable within an individual and different from that of others. Establishing the EEG as not only stable within an individual, but unique from that of others is crucial for establishing useful endophenotypes. Although stability studies have shown regional differences, studies using ICCs measures have not addressed regional issues beyond comparing central versus frontal derivations in children with and without dyslexia [16] and cerebral palsy [17], finding ICC values between these regions are roughly equivalent. A limitation of the previous research is that both ICC values and correlation coefficients are computed for a single frequency bin or band and therefore do not capture the features of the entire EEG power density spectrum.

The aim of the current study was to examine the stability of the *entire* waking EEG spectrum in early adolescents (ages 11 to 13 years) over weekly intervals and examine the extent to which the waking EEG spectrum is a biological trait in this age range. We used a cluster analysis based on distance between the three weekly-recorded power density spectra to assess whether the EEG spectrum is trait-like. Compared to ICC analysis, in cluster analysis the entire EEG spectrum is taken into account, making it more representative of an individual, thus enhancing its utility and validity. Unlike ICC, cluster analysis does not rely on a priori knowledge of repeated measures within a given individual and thus does not make use of intra and inter-individual variability. Rather, in cluster analysis, spectra are grouped according to their similarity. Furthermore, we examined whether trait-like characteristics, as determined by the cluster analysis, vary in central and occipital EEG derivations. In order to compare our results with previous stability studies, we also examined the stability of the alpha peak across recordings using an ANOVA.

## Methods

### A. Subjects

Twenty-two healthy right-handed early adolescents, aged 11 to 13 years (mean age 12.3 ± 0.8 years; 12 males) participated in this study. All participants were pre/early pubertal (n = 13) or mid-pubertal (n = 8) with the exception of one female who was late pubertal.

Participants were recruited using television and newspaper advertisements, as well as at schools, and at a meeting of the association for parents with gifted children. The Zurich cantonal ethical committee (KEK) for research on human participants approved the protocol and the participants’ legal guardian gave written informed consent. This study conforms with the Code of Ethics of the World Medical Association (Declaration of Helsinki), printed in the British Medical Journal (18 July 1964). Participants were compensated for their participation by cinema, book or CD vouchers and a T-shirt.

Participants were healthy, had no history of neurologic and psychiatric disease, and were medication free. For three days before each experimental session participants were asked to abstain from caffeine and medication and to adhere to a regular sleep–wake schedule (minimum of 8 hours of night-time sleep; no daytime naps). Wrist-worn actimeters and sleep logs verified compliance. The data used in the current study is the control data from another study [18].

### B. Study design

Participants came to the laboratory on three different occasions in weekly intervals, always at the same time of the day. At each session, a baseline waking EEG was recorded to assess the stability of waking EEG in early adolescents over weekly intervals.

### C. Electroencephalogram

EEG (derivations C3LM, C4LM, O1LM and O2LM; LM = Linked Mastoid), electrooculogram (EOG), and electrocardiogram (ECG) were recorded with a polygraphic amplifier Artisan (Micromed, Mogliano Veneto, Italy). The analog signals were high-pass (EEG: –3 dB at 0.16 Hz; ECG: 1 Hz) and low-pass filtered (−3 dB at 67.2 Hz), sampled at 256 Hz, and recorded using Rembrandt DataLab (Version 8.0; Embla Systems, Broomfield, CO, USA).

Waking EEG was recorded continuously for 6 min (3 min eyes closed, 3 min eyes open). Participants sat on a chair, rested their head on a chin rest, and were instructed to avoid movement. Vigilance was ensured by continuous online visual inspection of the recordings and alerting the subjects via intercom when signs of drowsiness were present.

### D. Data analysis

#### D1. Electroencephalogram

Artifacts were visually marked and artifact-free segments were subjected to spectral analysis (Hanning window of width 2 s with 50% overlap; frequency resolution of 0.5 Hz) using MATLAB (The MathWorks Inc, Natick, MA, USA). All participants had at least thirty 2-s epochs of artifact free data in each condition (i.e., eyes closed and open) which were used to calculate the power density spectra. Frequencies between 1 and 20 Hz were analyzed.

#### D2. Cluster analysis of power density spectra

**Figure 1:**
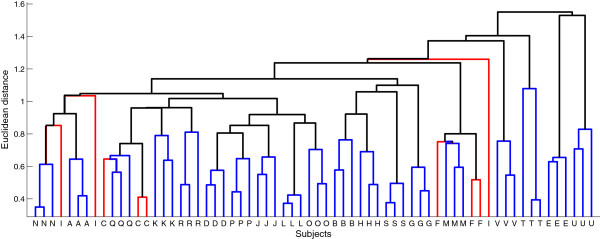
**Dendrogram of derivation C3LM in the eyes closed condition for illustrative purposes.** This analysis included data from 22 participants whose waking EEG was recorded in weekly intervals for three weeks. In this plot, each participant is denoted with an alphabetical letter on the x-axis and Euclidean distance is shown on the y-axis. Clusters highlighted in blue depict participants clustering across all three weeks, while red depicts participants whose three recordings do not cluster together. In this example, data from 19 participants clustered correctly, while data from 3 (subjects F, I, and C) did not cluster correctly.

Hierarchical cluster analysis based on Euclidean distance was used to examine whether the log-transformed EEG spectra (from 1 to 20 Hz) were trait-like using MATLAB functions PDIST and LINKAGE (average aggregation strategy). Cluster analysis was performed in several steps. First, a power spectrum was obtained for each recording/participant and represented as a vector consisting of power density at all frequency bins (e.g., 1 to 20 Hz with a frequency resolution of 0.5 Hz results in a vector of 39 values). Next, the distance between all vectors (total of 66 vectors = 22 participants multiplied by three spectra per participant) was calculated and vectors with small distances between them were clustered together, while vectors that were far apart were clustered separately. Finally, the distance between vectors is represented visually as a dendrogram, which consists of upside-down U-shape lines where the height of the U represents the distance between the connected objects (Figure 1). The cluster analysis was performed separately for each derivation, and ‘eyes open’ and ‘eyes closed’ conditions. The algorithm did not have a priori information regarding the number of clusters or recordings per participant; therefore clustering was based solely on distance. The percentage of correctly clustering participants was calculated for each derivation and ‘eyes open/closed’ conditions. We performed a χ^2^ test to assess whether the rate of correct clustering was significantly different between derivations or ‘eyes open/closed’ conditions.

#### D3. Stability of peak alpha frequency

To be able to compare our results with previous studies and investigate whether the characteristics of the alpha rhythm vary across sessions and derivations, we determined the frequency and height (measured in μV^2^/Hz) of the alpha peak. The alpha peak was determined on an individual basis because significant interindividual variability in the frequency of the alpha peak has been shown [19]. Three participants consistently showed more than a single peak in the alpha band (7–12 Hz). In these instances, the peak with the lower frequency was used for the analysis. Statistical analysis was performed with SPSS (Version 18.0; SPSS Inc, Chicago, IL) using a repeated measure ANOVA with factor ‘week’ (one, two, or three) to assess whether the frequency and height of the alpha peak differed across weeks. Analyses were done separately for eyes closed and open conditions since the effect of eye closure on the alpha peak has been well established [19,20]. Mauchly’s sphericity test was applied to all ANOVA tests and corrected using the Greenhouse-Geisser correction. Significant differences were further explored with post-hoc t-tests.

#### D4. Questionnaire data

A mood questionnaire using a 100 millimeter (mm) visual analog scale similar to Aitken [21], was administered at each session prior to the EEG recording. The questionnaire consisted of five questions (with the anchors indicated in parenthesis) regarding tiredness (0 mm = tired; 100 mm = alert), general mood (0 mm = good mood; 100 mm = bad mood), energy (0 mm = lethargic; 100 mm = energetic), tension (0 mm = relaxed; 100 mm = stressed), and concentration (0 mm = concentrated; 100 = unable to concentrate). A repeated measures ANOVA with factor ‘week’ (one, two, or three) was used to assess whether there was a change in these parameters across weeks. Significant effects were further explored with post-hoc t-tests comparing the weeks.

## Results

### A. Cluster analysis of power density spectra

**Figure 2:**
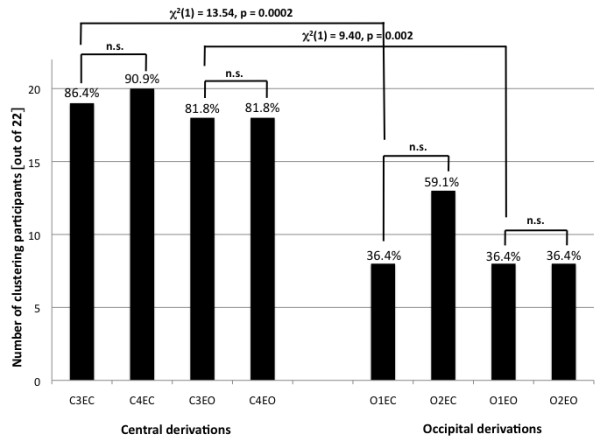
**Rate of clustering in central and occipital derivations.** Rate of clustering is depicted for central and occipital derivations. Each bar represents the results of the cluster analysis for a given derivation in the eyes open and closed conditions with the percentage of participants who cluster correctly noted on top. The y-axis shows the number of participants who cluster correctly. Significant differences (p<0.05; n.s., non-significant) were analyzed using a χ^2^ test.

In order to compare the morphology of the entire spectrum we performed a hierarchical cluster analysis (see Methods). The number of participants that clustered successfully over all three recordings was dependent on EEG derivation (Figure 2). For the central derivations in the eyes closed condition, 19 participants clustered for C3LM and 20 participants clustered for C4LM. The dendrogram for the eyes closed condition for derivation C3LM is shown in Figure 1. For the eyes open condition, the rate of clustering was the same for C3LM and C4LM (18 of 22 participants). In contrast, for O1LM and O2LM clustering was successful in only 8 participants in the eyes open condition. In the eyes closed condition, clustering was successful in 13 participants for O2LM and 8 participants for O1LM.

**Figure 3:**
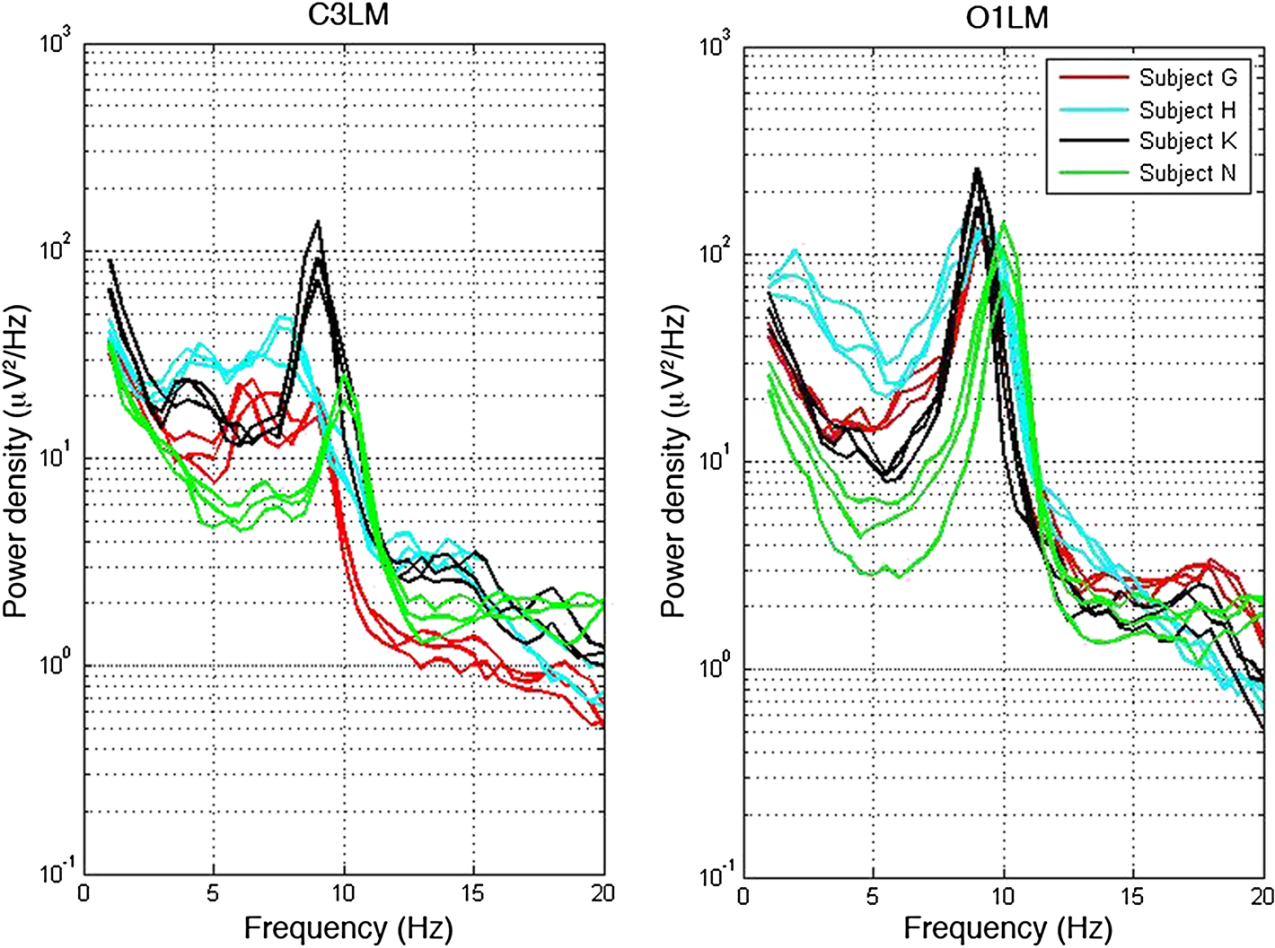
**Comparison of the power spectra in participants whose recordings cluster in C3LM but not in O1LM.** Power density spectra of four representative participants are shown for derivation C3LM (left) and O1LM (right). Each participant was recorded three times resulting in three spectra (depicted in one color) per participant. The four participants shown clustered across all the three recordings for derivation C3LM but not for derivation O1LM. Note that power density is plotted on a logarithmic scale.

Chi-squared analyses revealed that clustering was more successful for the left central compared to the left occipital derivation (Eyes closed: χ^2^(1) = 13.54, p = 0.0002; Eyes open: χ^2^(1) = 9.40, p = 0.002). To demonstrate this difference, four participants whose spectra clustered in the eyes closed condition for derivation C3LM but not O1LM are shown in Figure 3. We did not observe a difference in clustering between right and left central (Eyes closed: χ^2^(1) = 0.23, p = 0.63, Eyes open: χ^2^(1) = 0, p = 1) or left and right occipital (Eyes closed: χ^2^(1) = 2.28, p = 0.13, Eyes open: χ^2^(1) = 0, p = 1) derivations (Figure 2).

### B. Peak alpha frequency and height

**Figure 4:**
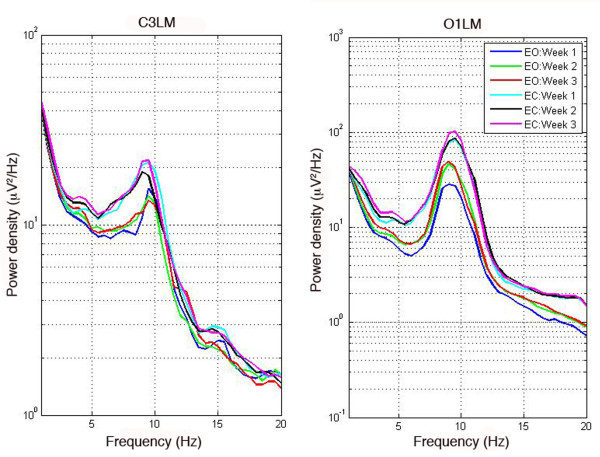
**Average power density spectra for derivations C3LM and O1LM.** Power density spectra averaged across participants of waking EEG were calculated for derivations C3LM (left) and O1LM (right) for three recording sessions separated by a week. Eyes open (EO) conditions are shown with blue, red, and green lines and eyes closed (EC) are shown in cyan, black, and magenta. Note that power density is plotted on a logarithmic scale and the scales are different for the two derivations.

We examined the stability of the peak height (power density measured in μV^2^/Hz) and frequency (Hz) of the alpha peak in the waking EEG spectrum using an ANOVA with factor ‘week’ (one, two or three). Average power spectra are shown in Figure 4 for left central and occipital derivations as a function of week and eyes open/closed. We found a modest change in the frequency of the alpha peak at the left occipital derivation (O1LM) for both eyes closed (F(2,42) = 4.94; p = 0.012) and open (F(2,42) = 5.4; p = 0.008) conditions. A decline in frequency in the eyes closed condition between weeks one and three (t(21) = 2.61; p = 0.016) but not between weeks one and two (t(21) = 0.81; p = 0.427) was observed. In the eyes open condition, we found a slowing of frequency between weeks one and two (t(21) = 2.11; p = 0.047) and weeks one and three (t(21) = 3.46; p = 0.002). We found no change in the frequency of the peak in the central derivations.

With regards to peak height of the alpha peak, we found a significant main effect of week for the occipital derivations in the eyes open condition only (O1LM: F(2,42) = 7.86; p = 0.001; O2LM: F(2,42) = 7.134, p = 0.006). Peak height increased between weeks one and two (O1LM: t(21) = −2.68; p = 0.014; O2LM: t(21) = −2.78; p = 0.011) and between weeks one and three (O1LM: t(21) = −4.12; p <0.001; O2LM: t(21) = −2.9; p = 0.009). Means and standard deviations for peak height and frequency are shown in Table 1 for all derivations for both eyes open and closed conditions.

**Table 1:** Descriptive data of the EEG alpha peak

**Derivation**	**Eyes closed**			**Eyes open**		
	**Week 1**	**Week 2**	**Week 3**	**Week 1**	**Week 2**	**Week 3**
**Frequency [Hz]**						
C3LM	9.2 (±1.2)	9.2 (±1.1)	9.2 (±1.2)	9.3 (±1.3)	9.1 (±1.1)	9.4 (±1.3)
C4LM	9.2 (±1.1)	9.2 (±1.2)	9.2 (±1.2)	9.4 (±1.2)	9.1 (±1.2)	9.3 (±1.1)
O1LM	9.7 (±0.7)	9.6 (±0.8)	9.5 (±0.7)	9.7 (±0.7)	9.5 (±0.7)	9.4 (±0.8)
O2LM	9.6 (±0.8)	9.5 (±0.8)	9.5 (±0.7)	9.5 (±0.8)	9.5 (±0.8)	9.4 (±0.7)
**Height [μV**^**2**^**/Hz]**						
C3LM	35.20 (±35.95)	28.69 (±20.19)	32.25 (±19.35)	24.49 (±22.07)	20.47 (±16.01)	22.11 (±15.79)
C4LM	34.39 (±34.83)	30.85 (±21.26)	30.78 (±17.47)	20.99 (±17.54)	20.78 (±14.58)	25.38 (±23.16)
O1LM	140.57 (±111.26)	137.20 (±95.46)	158.81 (±127.88)	44.91 (±57.47)	63.38 (±61.77)	70.65 (±77.38)
O2LM	147.27 (±128.18)	138.68 (±127.12)	165.17 (±134.02)	49.05 (±60.47)	71.57 (±86.89)	75.57 (±90.50)

Mean (standard deviation) frequency (Hz) and height (μV^2^/Hz) of alpha peak (7 – 12 Hz). Frequency of the peak declined in the eyes open and closed condition between weeks one and three in the left occipital derivation (O1LM). Peak height increased in the occipital derivation (O1LM and O2LM) across weeks in the eyes open condition.

### C. Mood questionnaire

A change in mental tension across weeks was observed (mean (SD): week 1 = 30.3 (16.8); week 2 = 29.7 (24.2); week 3 = 27.6 (21.1); F(2,42) = 4.248; p = 0.036). A post-hoc paired t-test revealed a decline in mental tension between weeks one and three (t(21) = 2.37; p = 0.028) but not between weeks one and two (t(21) = 1.88; p = 0.075). No other variables showed a significant change across weeks.

### D. Impact of mood on alpha peak characteristics

We included mood as a covariate in the ANOVA analysis in order to examine whether the change in mental tension had an impact on the observed effects in the occipital derivations. Examining the data in this way, there was no longer an effect of week for O1LM peak frequency in the eyes closed condition and O2LM peak height in the eyes open condition.

## Discussion

The current study used two different measures to examine the stability of the EEG spectrum in early adolescents: a cluster analysis approach and characterization of the alpha peak. Overall, the results showed that the waking EEG is highly stable across recordings and unique to an individual. In addition, we were able to demonstrate that the degree to which the EEG spectrum is trait-like is dependent on the brain region.

### Clustering is better in central derivations

The current study used cluster analysis to examine whether the waking EEG in early adolescents represents a trait. Thus, the unique contribution of our analysis is not solely the examination of stability, but also the quantification of interindividual variability. Another advantage of the current analysis is that clustering is based on the *entire spectrum* rather than limited to the alpha band. Although the alpha oscillation comprises an important cortical rhythm, functional neuroanatomy is more accurately reflected in the entire EEG spectrum.

Our analysis showed that central, and to a lesser extent, occipital derivations, are trait-like. We note that though the percentage of clustering for occipital derivation appears low (between 36.4% and 59.1%), we would not expect successful clustering for any participant by chance. We do not expect that the difference between central and occipital derivations is due to less stability of the alpha peak in the occipital regions because our ANOVA analysis revealed a change in alpha peak characteristics in only the eyes open condition, whereas the difference between occipital and central derivations was present in both eyes open and closed conditions. The lack of *inter*individual variability rather than low *intra*individual stability may account for the lower rate of clustering in occipital versus central derivations. For example, greater variability across individuals was observed in the frequency and shape of the waking EEG spectrum in central compared to occipital derivations (see Figure 3). We speculate that in functionally “lower” cortical areas (e.g., occipital regions) variability across individuals is limited since these regions perform the first steps in processing of visual stimuli, which tends to occur in a stereotypical manner [22]. On the other hand, cortical regions that are involved in further processing of stimuli and coordination of action (e.g., central regions) involve many connections and may be more dependent on individual differences in neuroanatomy [23].

### Clustering in sleep versus waking

In a previous study of trait-like characteristics of the sleep EEG in a different sample of adolescents, Tarokh et al. [24] found that successful clustering of non-rapid eye movement sleep EEG spectra recorded on consecutive nights at derivation C3A2 was comparable to that found in the current study. In contrast to the current study, however, the study by Tarokh et al. found similar rates of successful clustering in the right occipital (O2A1) and left central (C3A2) derivations (i.e., 89% in O2A1 and 94% in C3A2). We speculate that the difference between clustering during waking and sleep may reveal important information regarding the brain oscillations present during these two states. During waking, occipital and central regions are functionally distinct with occipital regions performing visual processing while central regions are involved in somatosensory information processing and execution of action. In contrast, during sleep, the functional difference between these areas may be less pronounced and cortical oscillations in these regions are less idiosyncratic.

### Analysis of the alpha frequency peak

The alpha frequency band is an important rhythm to consider when examining the heritability and stability of the waking EEG. Several studies have shown that alpha peak power is a highly stable EEG parameter [11] and that heritability is highest around the alpha peak [3]. In the current study, no significant changes in alpha peak frequency or peak height across weeks were found in central EEG derivations. This adds further support to previous reports showing that the alpha rhythm in children has high test-retest reliability [12,14,16]. In contrast, analysis of occipital derivations across weeks showed significant changes in both the frequency and height of the alpha peak. This finding is in contrast to previous studies that found greater stability over more anterior regions. Compared to previous studies the time interval between assessments was short (one week) in the current study. Therefore, we interpret our results in conjunction with the mood questionnaires, which showed a decline in mental tension across weeks. Alpha is prominent over occipital regions and reflects the degree of relaxation – as one increases so does the other. Therefore, the changes to alpha peak height and frequency across weeks may reflect an adaptation of participants to the lab environment and study protocol over time. This adaptation may not occur in studies where recordings were several weeks or months apart. Therefore, our finding highlights the importance of including a baseline EEG recording at every experimental session in order to ensure accuracy of results and avoid unnecessary inflation of Type I error.

### Limitations

Several limitations of this study are important to note. With respect to our analysis of the stability of the alpha frequency peak, our frequency resolution was 0.5 Hz, which limits our ability to detect more subtle changes in frequency across recording session. Furthermore, this analysis was restricted to four derivations, which limits our ability to examine regional differences in further detail. We examined a narrow age range and the degree to which the regional differences we observe are specific to this developmental stage is unknown. In fact, there are large differences in the maturational trajectory of different cortical regions [25]. Future studies should examine the degree to which the EEG is trait-like using a larger number of electrodes and more participants.

## Conclusions

We used a novel method to show not only that the waking EEG spectrum is trait-like, but that the degree to which the EEG is trait-like depends on brain region. This finding has implications for resting state waking EEG studies in search of biological markers of cognitive capabilities and psychiatric disorders suggesting that such studies should use central rather than occipital derivations.

## Competing interests

The authors have no competing interests to declare.

## Authors’ contributions

SPL and PA designed the study. LT, SPL and DB performed data analysis. DB and SPL participated in data collection. LT, SPL, DB and PA wrote the manuscript. All authors read and approved the final manuscript.

## Authors’ information

Peter Achermann, Sarah P Loughran Shared senior authorship.

Dominik C Benz, Leila Tarokh Shared first authorship.
